# 

**Published:** 1844-04

**Authors:** 


### CONTENTS OF No. XXXIV

OF THE

### British and Foreign Medical Review.

APRIL, 1844.

---

### Part First.—Analytical and Critical Reviews.

PAGE

Art. I.—Traité Clinique et Pratique des Maladies des Enfans. Par MM. Rilliet et Barthez . . . . . . . 293

A Clinical and Practical Treatise on the Diseases of Children. By MM. Rilliet and Barthez.

Art. II.—1. Statistical Reports of the Sickness, Mortality, and Invaliding among the Troops. Compiled by Major Tulloch, and presented to Parliament, 1838-41 . . . . . . . . 313

2. Statistical Reports on the Health of the Navy, from 1830 to 1836. Compiled by Dr. Wilson, and ordered by the House of Commons to be printed . ib.

Art. III.—Cataract, and its Treatment; comprising an easy mode of dividing the Cornea for its extraction, and appropriate means for removing the different forms of that affection. By John Scott, Senior Surgeon to the Royal London Ophthalmic Hospital . . . . 329

Art. IV.—Recherches Expérimentales sur l'Inanition; Mémoire auquel l'Académie des Sciences a décerné, en 1841, le prix de Physiologie expérimentale. Par Charles Chossat, m.d., Membre de plusieurs sociétés savantes . 344

Experimental Researches on Inanition; the Memoir to which the Prize for Experimental Philosophy was adjudged by the Academy of Sciences in 1841. By Charles Chossat, m.d. &c.

Art. V.—The History, Diagnosis, and Treatment of Typhoid and Typhus Fever; with an Essay on the Diagnosis of Bilious Remittent and Yellow Fever. By Elisha Bartlett, m.d. Professor of Medicine in Transylvania University . . . . . . . 357

Art. VI.—Der weiche Hinterkopf; ein Beitrag zur Physiologie und Pathologie der ersten Kindheit. Von Dr. C. L. Elsaesser . . . 371

The Soft Occiput; a Contribution to the Physiology and Pathology of Early Childhood. By Dr. C. L. Elsaesser.

Art. VII.—1. Die Selbständigkeit des sympathischen Nervensystem durch anatomische Untersuchungen nachgewiesen. Von F. H. Bidder und A. W. Volkmann, Professoren in Dorpat . . . . 379

The Independence of the Sympathetic Nervous System, demonstrated by Anatomical Researches. By F. H. Bidder and A. W. Volkmann, Professors at Dorpat.

§

PAGE

2. Untersuchungen über den Bau den Nervensystems. Von Dr. B. STILLING und Dr. J. WALLACH. Erstes Heft, enthaltend Unter suchungen über die Textur des Rückenmarks. Zweites Heft, enthaltend Untersuchungen über die Textur und Function der Medulla Oblongata . . . 379

Researches into the Structure of the Nervous System. By Dr. B. STILLING and Dr. J. WALLACH. Part I—Researches into the Texture of the Spinal Cord. Part II—Researches into the Structure and Function of the Medulla Oblongata.

3. Traités et Découvertes sur la Physiologie de la Möelle Epinière. Par J. VAN DEEN, Docteur en Médecine, &c. &c. . . . . ib.

Treatises and Discoveries on the Physiology of the Spinal Cord. By J. VAN DEEN, M.D. &c.

4. Untersuchungen über die Functionen der Rückenmarks und der Nerven. Von Dr. B. STILLING, pract. Artze und Wundartze zu Cassel . . ib.

Inquiries into the Functions of the Spinal Cord and Nerves. By Dr. B. STILLING, Physician and Surgeon at Cassel.

5. Untersuchungen über das Nervensystem. Von Dr. JULIUS BUDGE . ib.

Inquiries respecting the Nervous System. By Dr. J. BUDGE.

6. Die Physiologie des Rückenmarks mit Beruchsichtigung seiner pathologischen Zustände für pratische Aertze. Von BENEDICT SCHULTZ, Doctor der Medicin ib.

The Physiology of the Spinal Cord, with reference to its Pathological States. By Dr. SCHULTZ.

7. Mikroskopiske Undersögelser of Nervesystemet. Ved ADOLPH HANNOVER, Lic. Med. . . . . . . . ib.

Microscopical Investigations of the Nervous System. By Dr. A. HANNOVER.

8. Untersuchungen über die Physiologie der Nervenfaser. Von Dr. GEORG HERMANN MEYER, Privatdozenten zu Tubingen, &c. . . ib.

Inquiries into the Physiology of Nervous Fibrils. By Dr. G. H. MEYER, Private Teacher at the University of Tubingen.

ART. VIII.—Report on the Sanitary Condition of the Labouring Population of Great Britain : a Supplementary Report on the results of a Special Inquiry into the Practice of Interment in Towns. By EDWIN CHADWICK, Esq., Barrister at Law. . . . . . . 403

ART. IX.—Lehrbuch des Nervenkrankheiten des Menschen. Von M. H. ROMBERG, Doctor der Medicin, &c. Erster Band . . . . 407

A Manual of the Nervous Diseases of Man. By M. H. ROMBERG, Doctor of Medicine, &c. Vol. I.

ART. X.—1. Traité de Chimie Pathologique, ou Recherches Chimiques sur les Solides et les Liquides du Corps Humain, dans leur rapports avec la Physiologie et la Pathologie. Par S. D. L'HERITIER . . . 424

A Treatise on Pathological Chemistry, or Chemical Researches on the Solids and Liquids of the Human Body, in their relations to Physiology and Pathology. By S. D. L'HERITIER.

2. Lehrbuch der Physiologischen Chemie. Von Dr. C. G. LEHMANN. 1ste Band ib.

A Treatise on Physiological Chemistry. By Dr. C. G. LEHMANN. Vol. I.

3. Anleitung zum Gebrauch des Mikroskopes zur Zoochemischen Analyse, und zur Mikroskopisch-chemischen Untersuchung uberhaupt. Von Dr. JULIUS VOGEL . . . . . . . . ib.

Introduction to the use of the Microscope in the Chemical Analysis of Animal Matters, and in Microscopico-chemical inquiries in general. By Dr. JULIUS VOGEL.

4. Chemistry of Animal Bodies. By THOMAS THOMSON, M.D. . . ib.

PAGE

ART. XI.—Œuvres Complètes d'Hippocrate, Traduction Nouvelle, avec le Texte Grec en regard, collationnés sur les Manuscrits et toutes les Editions ; accompagnée d'une Introduction, de Commentaires Médicaux, de Variantes et de Notes Philologiques; suivi d'une Table générale des Matières. Par E. LITTRÉ, Membre de l'Institut et de la Société d'Histoire Naturelle de Halle 450

The Complete Works of Hippocrates, a new Translation, with the Greek Text on the opposite Pages, collated with the Manuscripts and all the Editions; with an Introduction, Medical Commentaries, various Readings, and Philological Notes; followed by a general index of the Matter therein contained. By E. LITTRÉ, &c.

2. Hippocratis Nomine quæ circumferuntur Scripta ad Temporis Rationes disposuit CHRISTIANUS PETERSEN, in Gymnasio Hamburgensium Academico Philol. Class. Prof. P.; Pars Prior . . . . ib.

The Writings which commonly go under the Name of Hippocrates, chronologically disposed by CHRISTIAN PETERSEN, &c.

ART. XII.—Principles of Medicine ; comprising General Pathology and Therapeutics, and a brief general view of Etiology, Nosology, Semeiology, Diagnosis, and Prognosis. By CHARLES J. B. WILLIAMS, M.D. F.R.S., &c. . 472

ART. XIII.—A Dictionary of Practical Medicine; comprising General Pathology, the Nature and Treatment of Diseases, &c. &c. By JAMES COPLAND, M.D. F.R.S., &c. . . . . . . 503

ART. XIV.—Medicinisches Schriftsteller-Lexicon der jetz lebenden Aertzte, Wundärtze, Geburtshelfer, Apotheker, und Naturforscher aller gebildeter Völker. Von ADOLPH CARL PETER CALLISEN, Doctor der Medicin und Chirurgie, Professor an der Königl. Chirurgischen Academie zu Copenhagen, &c. . 504

Lexicon of Living Medical Authors, Physicians, Surgeons, Man-midwives, Apothecaries, and Natural Historians of all civilized nations. By A. C. P. CALLISEN, M.D.

ART. XV.—A Practical Treatise on Organic Diseases of the Uterus. By JOHN C. W. LEVER, M.D., Assistant Accoucheur at Guy's Hospital, &c. &c. . 506

ART. XVI.—Some Account of Cretinism, and the Institution for its Cure on the Abendberg, near Interlachen, in Switzerland. By W. TWINING, M.D. . 514

ART. XVII.—The Sources of Physical Science; being an Introduction to theStudy of Physiology through Physics, comprising the connexion of the several departments of Physical Science, their dependence on the same Laws, and the relation of the Material to the Immaterial. By ALFRED SMEE, F.R.S. Surgeon to the Bank of England, &c. . . . 517

ART. XVIII.—Beiträge zur Kenntniss der Kranken Schleimhaut der Respirationsorgane und ihrer Producte durch das Mikroskop. Von FR. BÜHLMANN . 519

Contributions to the Knowledge of the Microscopic Characters of the Mucous Membrane of the Respiratory Organs and its Products. By FR. BÜHLMANN.

ART. XIX.—The Epidemics of the Middle Ages. From the German of J. F. C. HECKER, M.D. Translated by B. G. BABINGTON, M.D. F.R.S. Printed for the Sydenham Society . . . . . . 522

### PART SECOND.—Bibliographical Notices.

ART. I.—Elements of Natural Philosophy, being an Experimental Introduction to the Study of the Physical Sciences. By GOLDING BIRD, A.M. M.D. F.L.S., &c. 523

ART. II.—Glossology, or the additional means of Diagnosis of Disease to be derived from Indications and Appearances of the Tongue. By BENJAMIN RIDGE, M.D. . . . . . . 524

PAGE

ART. III.—The Distinction between Instinct and Reason; the Introductory Discourse to a Series of Lectures on the Properties and Functions of Animal Life, delivered before the Philosophical and Literary Society at Portsmouth. By J. STRANG, M.D., Vice-president of the Society . . . 525

ART. IV.—Observations on the Proximate Cause of Insanity. By JAMES SHEPPARD, M.R.C.S., being an attempt to prove that Insanity is dependent on a Morbid Condition of the Blood . . . . . 526

ART. V.—Lectures on the Theory and Practice of Midwifery. By R. LEE, M.D. F.R.S. 527

ART. VI.—The Vital Statistics of Sheffield. By G. CALVERT HOLLAND, Esq. M.D. Physician extraordinary to the Sheffield General Infirmary, &c. . 528

ART. VII.—Pathological and Histological Researches on Inflammation of the Nervous Centres. By JOHN HUGHES BENNETT, M.D., &c. &c. . ib.

ART. VIII.—Medicines, their Uses and Modes of Administration; including a complete Conspectus of the three British Pharmacopœias, an Account of all the New Remedies, and an Appendix of Formulæ. By J. MOORE NELIGAN, M.D., Physician to the Jervis-street Hospital, &c. . . . 529

ART. IX.—An Anatomical Description of the Human Gravid Uterus and its Contents. By the late William Hunter, M.D. &c. Second Edition, by EDWARD RIGBY, M.D. &c. . . . . . . 530

ART. X.—The Principles of Physiology applied to the Preservation of Health, and to the Improvement of Physical and Mental Education. By ANDREW COMBE, M.D. &c. . . . . . . ib.

ART. XI.—On the Successful Treatment and Prevention of Consumption and Scrofula; Female Disorders connected therewith; Strumous Glandular Swellings, &c. By J. J. FURNIVALL, M.D. SECOND EDITION . . 531

ART. XII.—Illustrations of the Theory and Practice of Ventilation, with Remarks on Warming, Exclusive Lightning, and the Communication of Sound. By D. B. REID, M.D. F.R.S.E. . . . . . 532

---

### PART THIRD.—Original Reports and Memoirs.

I. Report on the Progress of practical Medicine in the Departments of Midwifery and the Diseases of Women and Children, during the years 1842-3. By CHARLES WEST, M.D., Member of the Royal College of Physicians, Physician to the Royal Infirmary for Children, and Physician-accoucheur to the Finsbury Dispensary. . . . . . 533

II. Report on the present State of our Knowledge of the Nature of Inflammation. By T. WHARTON JONES, F.R.S., Lecturer on Anatomy, Physiology, and Pathology at the Charing-Cross Hospital; Corresponding Member of the Imperial and Royal Society of Physicians and Surgeons at Vienna, &c. &c. 567

---

BOOKS RECEIVED FOR REVIEW . . . . . 586

TITLE, INDEX, &c.

